# Evaluation of drug information resources for interactions between therapeutic drugs and drugs of abuse

**DOI:** 10.5195/jmla.2020.969

**Published:** 2020-10-01

**Authors:** Robert D. Beckett, Jennifer R. Martin, Curtis D. Stump, Megan A. Dyer

**Affiliations:** 1 rdbeckett@manchester.edu, Drug Information Center, Manchester University, Fort Wayne, IN; 2 jenmartin@arizona.edu, Health Sciences Library, University of Arizona, Tucson, AZ; 3 CDStump2018@manchester.edu, Pharmacy Program, Manchester University, Fort Wayne, IN; 4 MADyer2018@manchester.edu, Pharmacy Program, Manchester University, Fort Wayne, IN

## Abstract

**Objective:**

The study evaluated point-of-care resources for scope, completeness, and consistency of information describing interactions between therapeutic drugs and drugs of abuse (DoA).

**Methods:**

A cross-sectional evaluation study was conducted focusing on seven resources: Clinical Pharmacology, Facts & Comparisons eAnswers, Lexicomp Online, Micromedex, *Drug Interactions Analysis and Management, Drug Interaction Facts,* and *Stockley's Drug Interactions.* A sample of clinically relevant interactions was developed through review of tertiary literature and resources, and input was solicited from subject matter experts. Entries from each resource for each interaction were evaluated for scope (i.e., whether there was an entry for the interaction); completeness (i.e., whether there was information addressing mechanism; clinical effects, severity, course of action, and level of certainty, described as a median rating on a 5-point scale); and consistency (i.e., whether the information in the resource was similar to the majority) among resources with an entry.

**Results:**

Following review by subject matter experts, the final sample contained 159 interactions. Scope scores ranged from 0.6% (*Drug Interactions Analysis and Management*) to 43.4% (Lexicomp Online). Completeness scores ranged from 2 (interquartile range [IQR] 0 to 3, *Stockley's Drug Interactions*) to 5 (IQR 5 to 5, *Drug Interaction Facts,* Micromedex, Facts & Comparisons eAnswers). Consistency scores ranged from 30.8% (*Stockley's Drug Interactions*) to 87.1% (Clinical Pharmacology) for severity and from 15.4% (Facts & Comparisons eAnswers) to 71.4% (*Drug Interaction Facts*) for course of action.

**Conclusions:**

Although coverage of drug-DoA interactions was low and content was often inconsistent among resources, the provided information was generally complete.

## INTRODUCTION

In 2018, 53.2 million Americans used illicit drugs, including cannabis, cocaine, crack cocaine, hallucinogens, heroin, inhalants, methamphetamine, and prescription stimulants [1]. Opioid misuse has been an epidemic in the United States, with 10.3 million users 12 years of age or older in the past year [2, 3]. Many drugs of abuse, whether legalized or not, possess clinically relevant interactions with therapeutic medications that could increase risk for adverse safety events or decrease therapeutic effectiveness of the prescribed drug [4]. Identifying and mitigating risk from these interactions is critical for protecting patient safety, and having appropriate, evidence-based information to guide health care professionals is essential.

Previous studies have evaluated content from tertiary drug information resources describing common drug-drug [5–8], drug-ethanol [9], and drug-tobacco interactions [9]. There have also been studies focusing on subgroups of drug-drug interactions involving psychiatric [10] and intensive care–related drugs [8]. However, none of these studies have addressed interactions between therapeutic drugs and drugs of abuse (DoA). The purpose of this study is to evaluate key point-of-care resources for scope and completeness and consistency of drug-DoA interaction information.

## METHODS

A cross-sectional evaluation study of seven tertiary, point-of-care drug information resources was conducted. Resources were selected based on a review of the American Association of Colleges of Pharmacy Library and Information Sciences guidance document, *Basic Resources for Pharmacy Education* [11], and for consistency with two similar published studies [7, 9]. The four commercially available electronic resources were Clinical Pharmacology powered by the ClinicalKey Drug Interaction Report (Clinical Pharmacology), Facts & Comparisons eAnswers Interactions (Facts & Comparisons), Lexicomp Online Interactions (Lexicomp Online), and IBM Micromedex Drug Interactions (Micromedex) [12–15]. The three print resources were *Drug Interactions Analysis and Management* (2014), *Drug Interaction Facts* (2015), and *Stockley's Drug Interactions* (eleventh edition) [16–18].

A sample of clinically relevant drug-DoA interactions was developed using a systematic PubMed search to yield results indexed to Medical Subject Headings (MeSH) for illicit drugs and drug interactions. Results were filtered to yield review articles. The search was conducted by an experienced pharmacy librarian. Articles were reviewed, and interactions that were described as clinically relevant were extracted. *Drugs of Abuse* (second edition) was also reviewed [4]. The list of potential interactions was shared with two subject matter experts: a neuropharmacologist and a board-certified clinical pharmacy specialist in psychiatry, both of whom teach in the DoA area. Subject matter experts were asked to review the list and recommend necessary additions or removals in order to generate a sample of the most clinically relevant interactions. Based on the methods of similar studies, a sample of 100 to 200 interactions was deemed to be sufficient for a valid assessment of the resources in terms of the end points. Interactions were selected if they resulted in potential safety concerns or decreased effectiveness of the therapeutic drug (as opposed to the DoA) [7, 9].

Data were extracted from each resource by two investigators using a standard, electronic data collection form, with any discrepancies resolved by consensus with a third investigator, who is a board-certified clinical pharmacy specialist in pharmacotherapy and a drug information specialist.

The following data points were gathered for each interaction from each resource: mechanism, potential clinical effects of the interaction, severity rating, recommended course of action, and level of certainty (Table 1) [7, 9]. Investigators coded severity as minor, moderate, major, or severe/contraindicated. Course of action was coded as no action; monitor, counsel, or inform; adjust dose or administration; or avoid concomitant use. The most serious severity rating or highest level course of action was used in the event that there were multiple ratings or courses of action for the same interaction.

**Table 1:** Collected data points and endpoints

		Data point	Endpoint
Scope		Entry for the interaction in the resource	Percentage of interactions with an entry
Completeness	Mechanism	Pharmacological explanation for how the interaction occurs	Percentage of interactions with unambiguous mechanism information
	Clinical effects of the interaction	Signs and/or symptoms indicative of the interaction	Percentage of interactions with unambiguous clinical effects information
	Severity rating	Potential seriousness of the interaction, if it occurs	Percentage of interactions with unambiguous severity information
	Recommended course of action	Recommended intervention to mitigate interaction risk	Percentage of interactions with unambiguous course of action information
	Level of certainty	Level of documentation supporting the interaction	Percentage of interactions with unambiguous level of certainty information
	Overall	1 point awarded for each completeness element, per interaction, per resource	Median score on a scale of 0 to 5 points
Consistency	Severity	Severity ratings, coded as minor, moderate, major, or severe/contraindicated	Percentage of interactions with the same severity coding as the majority of resources
	Course of action	Recommended course of action, coded as no action; monitor, counsel, or inform; adjust dose or administration; avoid concomitant use	Percentage of interactions with the same course of action coding as the majority of resources

Similar to previous studies, 3 key endpoints were assessed: scope, completeness, and consistency (Table 1) [7, 9]. Scope was defined as the percentage of interactions that possessed an entry in the resource. Completeness was defined for each data extraction element (i.e., mechanism, clinical effects, severity, course of action, level of certainty) as the percentage of interactions that contained the information. An overall completeness score was calculated by summing points for the 5 data extraction elements (i.e., 1 point per element, to yield a score of 0 to 5 possible points for each interaction, for each resource) and then calculating the median score by resource.

Consistency was defined as the percentage of interactions in each resource with content that was similar to the majority of resources. For example, if a resource rated an interaction as “major” in severity, and the majority of resources used the same rating, the first resource would be considered consistent with the rest. However, it would not be considered consistent if it rated the interaction as “minor.” Consistency was determined using severity and course of action, as the most objectively codable data extraction elements.

Descriptive statistics (percentage for nominal data, medians with interquartile range [IQR] for ordinal data) were primarily used to describe results for scope, completeness, and consistency. A tier analysis was also conducted to group resources by similar scope and completeness scores. The highest scoring resource was compared to the next highest scoring resource, serially, until the difference in score was statistically significant using a prespecified alpha value of 0.05. Once a difference was found to be statistically significant, the lower scoring resource formed the beginning of a new tier, and the process was continued in the same manner.

The McNemar test was used for scope scores (nominal data) and the Wilcoxon signed-rank test was used for completeness scores (ordinal data). Statistical tests for paired data were selected because the same sample of interaction pairs was used to evaluate each resource. Tier analysis was not conducted for consistency results, since two distinct consistency measures were considered (i.e., severity and course of action) and given missing data from instances when only one or two resources had entries for the information. Inferential statistics were conducted using IBM SPSS, version 24 [19].

## RESULTS

The initial literature search identified 169 drug-DoA interactions. Following review by subject matter experts, a final sample of 159 interaction pairs was formed. The primary reason for removal was if the interaction would have resulted in decreased “effectiveness” of the DoA (i.e., the therapeutic drug would have decreased the levels or activity of the DoA). The final sample, organized by DoA, is available in the supplemental appendix.

Of the 159 interaction pairs in the sample, 69 (43.4%) had an entry in Lexicomp Online, 68 (42.8%) had entries in Clinical Pharmacology and *Stockley's Drug Interactions*, 28 (17.6%) had an entry in Micromedex, 24 (15.1%) had an entry in Facts & Comparisons, 7 (4.4%) had an entry in *Drug Interaction Facts*, and 1 (0.6%) had an entry in *Drug Interactions Analysis and Management*. The scope tier analysis led to formation of 3 tiers: tier 1 (Lexicomp Online, Clinical Pharmacology, *Stockley's Drug Interactions*; *p*0.001 versus all remaining resources); tier 2 (Micromedex, Facts & Comparisons; *p*0.001 versus all remaining resources); and tier 3 (*Drug Interaction Facts, Drug Interactions Analysis and Management*).

Completeness results, in terms of the 5 specific completeness elements and overall completeness scores, are provided in Table 2. *Drug Interactions Analysis and Management* is not included in these results, as well as in the consistency results, since there was only a single interaction pair available from it for analysis (it provided information for mechanism, clinical effects, and course of action, but not severity or level of certainty). The completeness tier analysis led to formation of 4 tiers: tier 1 (*Drug Interaction Facts*, Micromedex, Facts & Comparisons; median 5, IQR 5 to 5; *p*0.001 versus all remaining resources); tier 2 (Lexicomp Online; median 4, IQR 4 to 5; *p*0.001 versus all remaining resources); tier 3 (Clinical Pharmacology; median 4, IQR 4 to 4; *p*=0.001 versus the remaining resource); and tier 4 (*Stockley's Drug Interactions*; median 2, IQR 0 to 3).

**Table 2:** Completeness elements and overall scores by resource

Resource	n	Mechanism*		Clinical effects*		Severity*		Level of certainty*		Course of action*		Completeness score†	
		n	(%)	n	(%)	n	(%)	n	(%)	n	(%)	n	range
Clinical Pharmacology	68	67	(98.5%)	67	(98.5%)	69	(100.0%)	3	(4.4%)	59	(86.8%)	4	(4 to 4)
*Drug Interaction Facts*	7	7	(100.0%)	7	(100.0%)	7	(100.0%)	7	(100.0%)	7	(100.0%)	5	(5 to 5)
Facts & Comparisons	24	23	(95.8%)	24	(100.0%)	24	(100.0%)	24	(100.0%)	24	(100.0%)	5	(5 to 5)
Lexicomp Online	69	38	(55.1%)	58	(84.1%)	69	(100.0%)	69	(100.0%)	69	(100.0%)	4	(4 to 5)
Micromedex	28	28	(100.0%)	28	(100.0%)	28	(100.0%)	28	(100.0%)	27	(96.4%)	5	(5 to 5)
*Stockley's Drug Interactions*	68	32	(47.1%)	32	(47.1%)	17	(25.0%)	12	(17.6%)	29	(42.6%)	2	(0 to 3)

* n (%); percentage of interaction pairs with clear, unambiguous information for the element.

† median (IQR); 1 point assigned for each completeness element; potential range 0 to 5 for each interaction pair.

Consistency scores are provided in Table 3. Consistency scores ranged from 30.8% (*Stockley's Drug Interactions*) to 87.1% (Clinical Pharmacology) for severity and from 15.4% (Facts & Comparisons) to 71.4% (*Drug Interaction Facts*) for course of action.

**Table 3:** Consistency scores by resource

Resource	Severity consistency*		Course of action consistency†	
	Score	(%)	Score	(%)
Clinical Pharmacology	54/62	(87.1%)	29/55	(52.7%)
*Drug Interaction Facts*	3/7	(42.9%)	5/7	(71.4%)
Facts & Comparisons	9/15	(60.0%)	2/13	(15.4%)
Lexicomp Online	54/65	(83.1%)	24/57	(42.1%)
Micromedex	10/24	(41.7%)	9/23	(39.1%)
*Stockley's Drug Interactions*	4/13	(30.8%)	6/14	(42.9%)

* Calculated as the percentage of available severity ratings for the resource that were similar to the majority of resources (e.g., out of 62 entries in Clinical Pharmacology with a severity rating, 54 (87.1%) were similar to the majority of the assessed resources).

† Calculated as the percentage of available course of action recommendations for the resource that were similar to the majority of resources (e.g., out of 55 entries in Clinical Pharmacology with a course of action rating, 29 (52.7%) were similar to the majority of the assessed resources).

## DISCUSSION

This study reported, for the first time, the quality of the information describing drug-DoA interactions available from standard, point-of-care, tertiary references. Overall, the scope of available information was low, with no resource having an entry for more than half of the interaction sample. However, the information that was available was generally very thorough. Completeness was highest for *Drug Interaction Facts*, Micromedex, and Facts & Comparisons.

Consistency was highly variable. Clinical Pharmacology and Lexicomp Online scored highest for consistency of severity ratings, while *Drug Interaction Facts* and Micromedex were most consistent for recommended course of action. Validity of results for completeness and consistency might be limited by the lower scope scores, which decreased the number of interactions available for analysis. *Drug Interaction Facts*, in particular, only had seven interactions available for analysis.

These results echo a previous study that examined drug-ethanol and drug-tobacco interactions [9]. For drug-ethanol interactions in particular, the resources that contained the most complete information were also Lexicomp, Micromedex, *Drug Interaction Facts*, and Facts & Comparisons. This same study identified Micromedex as providing the most complete information on drug-tobacco interactions and found similar overall results for completeness and scope of information. To date, no other studies have evaluated point-of-care resources or open access tools such as National Drug File: Reference Terminology (NDF-RT) and DrugBank for drug-DoA interactions [5–8, 10, 20].

Drug interaction detection software can detect harmful interactions and is available through several commercial sources. Some of the most commonly utilized commercial software among drug information experts are Micromedex, Lexicomp, and Facts & Comparisons [21, 22]. According to Grizzle et al., the most commonly used commercially available databases for detecting potential drug-drug interactions were Lexicomp and Micromedex, which were evaluated in a study [22]. Results from that study also revealed that other used resources included UpToDate Drug Interactions (containing the same information as Lexicomp), Epocrates, and Clinical Pharmacology, in addition to a few open access information resources such as Drugs@FDA and DailyMed [22]. This study did not evaluate Epocrates since it was not recognized in *Basic Resources in Pharmacy Education* [11]. This study did not use prescribing information from Drugs@FDA or DailyMed, because of their lack of a specific interaction checking tool and the importance of off-label information in evaluating interaction risk.

A limitation of this study was that only seven references were evaluated, and none were open access. However, the four primary commercially available drug information databases were represented, and selection of resources was based on expert guidelines and previously published studies [7, 9]. Based on previous surveys, the top preferred resources that would provide off-label information on drug-DoA interactions were evaluated in this study [21, 22]. It should be noted that differences in completeness scores were often driven by the level-of-certainty element. If a resource did not have a systematic heading that addressed this element, it might not have been as easy to detect the relevant content. Additionally, two resources, Clinical Pharmacology and *Stockley's Drug Interactions*, did not consistently provide a recommended course of action. Lack of this key element could hinder health care professionals' confidence in using the resource to aid them in clinical decision-making.

Results from this study can be used to help librarians and drug information specialists prioritize and justify subscriptions during times of scarce resources. They can also help improve efficiency when responding to questions from patrons, patients, or health care professionals or when recommending resources to these individuals. For example, it was anecdotally noted in these results that *Stockley's Drug Interactions* appeared to disproportionately cover Schedule I drugs, compared to other resources. Classroom or experiential instruction on drug interactions, from clinical or information mastery perspectives, can be greatly enhanced by the knowledge of which resources to use during specific situations. Future similar studies could continue to systematically explore drug interactions content by examining drug-food, drug-laboratory, drug-pregnancy, and drug-dietary supplement interactions. The quality of available information is also unknown for other types of drug information, such as pregnancy and lactation and off-label use content.

This study provides an overview of drug-DoA interactions of several key tertiary resources. Although coverage of drug-DoA was low and content was often inconsistent among resources, the provided information was generally complete. Overall, these results can be used in education, collection management, and clinical practice to help guide users to the best resources based on the information that is needed.

## Data Availability

Data from this study can be located in Open Science Framework at https://osf.io/m79v6.

## SUPPLEMENTAL FILE

**Appendix:** Sample of drug–drugs of abuse (DoA) interaction pairs
