# Supplementary material for: Evaluation of drug information resources for interactions between therapeutic drugs and drugs of abuse
Source: J Med Libr Assoc. 2020 Oct 1;108(4):584–90. doi: 10.5195/jmla.2020.969 (PMC7524612; doi:10.5195/jmla.2020.969)
Supplement: Supplementary file 1 — Appendix: Sample of drug–drugs of abuse (DoA) interaction pairs [file jmla-108-4-584-s01.pdf]

JMLA Journal of the Medical Library Association

SC
Supplemental content to
J Med Libr Assoc. Oct;108(4):dx.doi.org/10.5195/jmla.2020.969
www.jmla.mlanet.org
© Beckett, Martin, Stump, Dyer 2020

Evaluation of drug information resources for interactions between therapeutic drugs and drugs of abuse

Robert D. Beckett; Jennifer R. Martin; Curtis D. Stump; Megan A. Dyer

APPENDIX

Sample of drug–drugs of abuse (DoA) interaction pairs

Drug of abuse	Interacting drug
Alprazolam	Cobicistat
Alprazolam	Ritonavir
Cannabis	Amitriptyline
Cannabis	Amobarbital
Cannabis	Amoxapine
Cannabis	Amprenavir
Cannabis	Atazanavir
Cannabis	Butabarbital
Cannabis	Citalopram
Cannabis	Clozapine
Cannabis	Cyclobenzaprine
Cannabis	Darunavir
Cannabis	Desipramine
Cannabis	Disulfuram
Cannabis	Doxepin
Cannabis	Duloxetine
Cannabis	Escitalopram
Cannabis	Fluoxetine
Cannabis	Fluvoxamine
Cannabis	Fosamprenavir
Cannabis	Haloperidol
Cannabis	Imipramine
Cannabis	Indinavir
Cannabis	Lithium
Cannabis	Lopinavir/ritonavir
Cannabis	Mexiletine
Cannabis	Nabumetone
Cannabis	Naproxen
Cannabis	Nortriptyline
Cannabis	Olanzapine
Cannabis	Paroxetine

JMLA Journal of the Medical Library Association

SC
Supplemental content to
J Med Libr Assoc. Oct;108(4):dx.doi.org/10.5195/jmla.2020.969
www.jmla.mlanet.org
© Beckett, Martin, Stump, Dyer 2020

Drug of abuse	Interacting drug
Cannabis	Pentobarbital
Cannabis	Protriptyline
Cannabis	Riluzole
Cannabis	Saquinavir
Cannabis	Secobarbital
Cannabis	Sertraline
Cannabis	Tacrine
Cannabis	Theophylline
Cannabis	Tipranavir
Cannabis	Tizanidine
Cannabis	Triamterene
Cannabis	Trimipramine
Cannabis	Zileuton
Cannabis	Zolmitriptan
Chlordiazepoxide	Cobicistat
Chlordiazepoxide	Ritonavir
Clonazepam	Cobicistat
Clonazepam	Ritonavir
Cocaine	Acebutolol
Cocaine	Amitriptyline
Cocaine	Amprenavir
Cocaine	Atazanavir
Cocaine	Atenolol
Cocaine	Betaxolol
Cocaine	Bisoprolol
Cocaine	Carvedilol
Cocaine	Clarithromycin
Cocaine	Clomipramine
Cocaine	Cyclosporine
Cocaine	Darunavir
Cocaine	Dihydroergotamine
Cocaine	Duloxetine
Cocaine	Efavirenz
Cocaine	Erythromycin
Cocaine	Etravirine
Cocaine	Fluoxetine
Cocaine	Fosamprenavir
Cocaine	Haloperidol
Cocaine	Imipramine

SC
Supplemental content to
J Med Libr Assoc. Oct;108(4):dx.doi.org/10.5195/jmla.2020.969
www.jmla.mlanet.org
© Beckett, Martin, Stump, Dyer 2020

Drug of abuse	Interacting drug
Cocaine	Indinavir
Cocaine	Labetalol
Cocaine	Lopinavir/ritonavir
Cocaine	Metoprolol
Cocaine	Nadolol
Cocaine	Nevirapine
Cocaine	Paroxetine
Cocaine	Phenelzine
Cocaine	Pindolol
Cocaine	Propafenone
Cocaine	Propranolol
Cocaine	Quinidine
Cocaine	Rasagiline
Cocaine	Risperidone
Cocaine	Saquinavir
Cocaine	Selegiline
Cocaine	Sirolimus
Cocaine	Sotalol
Cocaine	St. John's wort
Cocaine	Tacrolimus
Cocaine	Telithromycin
Cocaine	Thioridazine
Cocaine	Timolol
Cocaine	Tipranavir
Diazepam	Cobicistat
Diazepam	Ritonavir
Fentanyl	Amiodarone
Fentanyl	Aripiprazole
Fentanyl	Baclofen
Fentanyl	Dabrafenib
Fentanyl	Olanzapine
Fentanyl	Quetiapine
Fentanyl	Risperidone
Gamma hydroxybutyrate	Eszopiclone
Gamma hydroxybutyrate	Zaleplon
Gamma hydroxybutyrate	Zolpidem
Gamma hydroxybutyrate	Zopiclone
Heroin	Cyclizine

JMLA Journal of the Medical Library Association

SC
Supplemental content to
J Med Libr Assoc. Oct;108(4):dx.doi.org/10.5195/jmla.2020.969
www.jmla.mlanet.org
© Beckett, Martin, Stump, Dyer 2020

Drug of abuse	Interacting drug
Heroin	Rasagiline
Heroin	Selegiline
Ketamine	Amobarbital
Ketamine	Butabarbital
Ketamine	Clarithromycin
Ketamine	Cobicistat
Ketamine	Efavirenz
Ketamine	Guanethidine
Ketamine	Nevirapine
Ketamine	Pentobarbital
Ketamine	Phenelzine
Ketamine	Procarbazine
Ketamine	Protriptyline
Ketamine	Rifampicin
Ketamine	Ritonavir
Ketamine	Secobarbital
Ketamine	Selegiline
Ketamine	Tranylcypromine
Ketamine	Trimipramine
MDMA	Amprenavir
MDMA	Atazanavir
MDMA	Darunavir
MDMA	Fluoxetine
MDMA	Fosamprenavir
MDMA	Indinavir
MDMA	Lopinavir/ritonavir
MDMA	Paroxetine
MDMA	Phenelzine
MDMA	Rasagiline
MDMA	Ritonavir
MDMA	Saquinavir
MDMA	Selegiline
MDMA	Tipranavir
Morphine	Metformin
Morphine	Metoclopramide
Morphine	Mexiletine
Morphine	Quinidine

MDMA: methylenedioxymethamphetamine.
